# Branched tetrameric lactoferricin peptides modified with diaminopropionic acid exhibit potent antimicrobial and wound-healing activities

**DOI:** 10.3389/fphar.2025.1719557

**Published:** 2025-12-02

**Authors:** Nikitha Vavilthota, Gizem Babuççu, Robert A. Cordfunke, Leonie de Boer, Payal Balraadjsing, Bouke K. H. L. Boekema, Jan W. Drijfhout, Martijn Riool, Sebastian A. J. Zaat

**Affiliations:** 1 Department of Medical Microbiology and Infection Prevention, Amsterdam Institute for Immunology and Infectious Diseases, Amsterdam University Medical Centre, University of Amsterdam, Amsterdam, Netherlands; 2 Department of Immunology, Leiden University Medical Centre, Leiden, Netherlands; 3 Burn Research Lab, Alliance of Dutch Burn Care, Beverwijk, Netherlands; 4 Department of Plastic, Reconstructive and Hand Surgery, Tissue Function and Regeneration, Amsterdam Movement Sciences Research Institute, Amsterdam University Medical Centre, Amsterdam, Netherlands; 5 Laboratory for Experimental Trauma Surgery, Department of Trauma Surgery, University Hospital Regensburg, Regensburg, Germany

**Keywords:** antimicrobials, wound healing, *Staphylococcus aureus*, *Candidozyma auris*, chronic wounds, bovine lactoferricin, *Ex vivo* wound model, multidrug-resistant (MDR)

## Abstract

**Introduction:**

Infected chronic wounds present a dual therapeutic challenge, requiring both the eradication of pathogens and the restoration of tissue homeostasis. Often the current treatments are ineffective against multidrug-resistant (MDR) pathogens and fail to promote wound healing. Antimicrobial peptides, such as bovine lactoferricin (LfcinB), offer a promising alternative owing to their broad-spectrum activity and immunomodulatory properties. The branched tetrameric LfcinB-derived peptide (LBT; (RRWQWR)_4_K_2_Ahx_2_-C_2_) is particularly attractive, as its multivalent architecture enhances antimicrobial potency and provides a tunable branching core for structural modifications.

**Methods:**

In this study, three novel tetrameric variants were designed by substituting the L-lysine branching residues in LBT with non-natural lysine derivatives to alter motif orientation and linker flexibility. Among the novel peptides, the diaminopropionic acid (DAP)-modified variant, (LBT-1; (RRWQWR)_4_DAP_2_Ahx_2_-C_2_) was selected as best-performing candidate based on antimicrobial and hemolytic activity assessment.

**Results:**

Compared to LBT, the novel LBT-1 demonstrated superior activity against methicillin-resistant *Staphylococcus aureus* (MRSA) and MDR *Acinetobacter baumannii*, achieving rapid bactericidal action within 5 minutes. LBT-1 also exhibited potent activity across the ESKAPE(E) panel and against the emerging MDR fungal pathogen *Candidozyma auris*. Beyond direct antimicrobial effects, LBT-1 enhanced macrophage-mediated bacterial clearance, neutralized endotoxins, and accelerated wound closure *in vitro*. Importantly, LBT-1 showed superior pro-angiogenic activity *in vitro* and achieved significantly higher bactericidal activity against MRSA in an *ex vivo* human skin wound infection model. This study identifies LBT-1 as a multifunctional therapeutic that addresses key pathological features of chronic wounds.

**Conclusion:**

Together, these findings validate our peptide design strategy, revealing previously unknown characteristics of the LBT peptide and the enhanced multifunctionality achieved with LBT-1, supporting its continued development for chronic wound management.

## 1 Introduction

Wound healing is an intricate physiological process encompassing four overlapping phases: hemostasis, inflammation, proliferation, and remodeling, requiring precise coordination among multiple cell types (Wilkinson and Hardman, 2020). However, this delicate orchestration can be disrupted by intrinsic factors (e.g., diabetes, vascular insufficiency) and extrinsic challenges (e.g., infections, biofilm formation), leading to chronic wounds (Frykberg and Banks, 2015). Chronic wounds, predominantly venous leg ulcers, pressure ulcers, and diabetic foot ulcers, represent a major clinical challenge due to their failure to progress through the normal healing phases in a timely manner (Falanga et al., 2002). The primary obstacle in chronic wound management is persistent infection that traps immune cells in a prolonged inflammatory state, generating excessive reactive oxygen species and proteolytic enzymes that prevent progression to the proliferative healing phase (Zhao et al., 2013; Wang et al., 2023). Chronic wounds are frequently colonized by *Staphylococcus aureus*, *Pseudomonas aeruginosa*, *Acinetobacter baumannii*, *Enterococcus faecalis*, and fungal species such as *Candida albicans*, which further complicate wound healing and treatment outcomes (Gjødsbøl et al., 2006; Ge and Wang, 2023). This problem is further exacerbated by infections caused by antibiotic-resistant bacteria and antifungal-resistant fungi, which limit treatment options and contribute to the growing global crisis of antimicrobial resistance (Siddiqui and Bernstein, 2010).

Antimicrobial peptides (AMPs) have emerged as promising alternatives to conventional antimicrobials due to their diverse mechanisms of actions, including membrane disruption, inhibition of protein synthesis, and interference with microbial metabolism (Zhang et al., 2021). These short peptides are key components of the innate immune system, providing rapid, non-specific defense against invading pathogens (Hancock, 1997; Huan et al., 2020). Beyond their broad-spectrum activity against drug-resistant pathogens, naturally occurring AMPs, also known as host defense peptides (HDPs), exhibit wound-healing and immunomodulatory functions, making them attractive multifunctional candidates for treating complex wound environments (Zhang and Gallo, 2016). However, clinical translation remains largely limited due to insufficient stability under physiological conditions and moderate membrane selectivity. To address these limitations of HDPs, branched peptide architectures have been developed (Meogrossi et al., 2024; Bracci et al., 2003; Falciani et al., 2012).

Bovine lactoferricin (LfcinB) derivatives are particularly noteworthy among HDPs for their multifunctional antimicrobial and immunomodulatory properties (Yami et al., 2023). The minimal antimicrobial motif within LfcinB is the hexapeptide sequence RRWQWR (Schibli et al., 1999). Building upon this core motif, peptide I.4, a branched tetrameric construct ((RRWQWR)_4_K_2_Ahx_2_C_2_) with lysine (K) residues at the branching core, demonstrated superior antimicrobial activity against MDR bacteria compared to its monomeric and dimeric counterparts (León-Calvijo et al., 2015). The branching core offers a tunable structural element, where modifications at this site can re-orient the spatial arrangement of the RRWQWR motifs, potentially optimizing peptide–pathogen interactions and expanding functional versatility for wound healing applications. Despite the potent antibacterial activity of I.4, its wound healing and immune-modulatory functions remain largely uncharacterized, both of which are critical for effective chronic wound therapy. In this study we designed novel tetrameric variants of I.4 and characterized their antimicrobial, immunomodulatory and wound healing capacity.

Here, the parent peptide 1.4 is designated as LfcinB tetramer (LBT) and three novel I.4 tetrameric variants were designed by substituting the two lysine residues at the branching core with unnatural lysine derivatives: 2,3-diaminopropionic acid (DAP; LBT-1) to reduce linker flexibility through shorter side chains (Stach et al., 2012); (2-(1,3-bisamino)propan-2-yl)oxy)acetic acid (DAPOA; LBT-2) to introduce steric bulk and hydrophobicity; and D-lysine (DK; LBT-3) to alter stereochemistry. Among the novel peptides, the best-performing candidate was identified through screening for antimicrobial activity and hemolysis assessment. Subsequently, LBT and the best-performing novel tetramer, were further tested against MDR bacterial and fungal strains relevant to chronic wound infections, as well as for bactericidal kinetics. Additional studies assessed cytocompatibility in skin cells, macrophage-mediated intracellular bacterial killing, and endotoxin neutralization. The wound healing potential of the peptides were investigated through *in vitro* scratch and angiogenesis assays. Finally, the antimicrobial efficacy was validated in an *ex vivo* human skin wound infection model against methicillin-resistant *S. aureus* (MRSA).

## 2 Results

### 2.1 Linker modifications influence selectivity index

To evaluate the impact of linker modifications on the selectivity index, both antimicrobial and hemolytic activities of the tetrameric peptides were assessed, using SAAP-148 and LL-37 as references. Antimicrobial activity was measured against the Gram-positive *S. aureus* and Gram-negative *A. baumannii* strains using the LC99.9 assay (Table 1). All peptides demonstrated potent bactericidal activity, with LC99.9 values <1 µM against both bacterial species. The presence of 50% plasma did not affect activity against *S. aureus,* but caused a four-fold increase in LC99.9 values against *A. baumannii*, indicating a partial reduction in activity.

**TABLE 1:** Hemolytic activity, bactericidal activity (LC99.9), and selectivity indices of LBT and variants, and of reference peptides (SAAP-148 and LL-37) in RPMI or 50% plasma against *Staphylococcus aureus* and *Acinetobacter baumannii*. Peptides were tested in a two-fold serial dilution range of 120–0.12 µM.

Peptides	Hemolytic^a^ concentration (HC)	Gram-positive		Gram-negative		Selectivity index^c^	
		*Staphylococcus aureus* JAR060131		*Acinetobacter baumannii* RUH875		HC_(µM)_/LC99.9 _(µM)Gm_ ^d^	
		RPMI	50% plasma	RPMI	50% plasma	RPMI	50% plasma
< 30% (µM)		LC99.9 _(µM)_ ^b^		LC99.9 _(µM)_			
LBT	>60	0.47	0.47	0.47	1.88	>128	>51
		(0.23-0.47)	(0.23-0.47)	(0.47-0.94)			
LBT-1	>60	0.47	0.47	0.47	1.88	>128	>51
		(0.23-0.47)	(0.47-1.88)	(0.47-0.94)	(1.88-3.75)		
LBT-2	60	0.47	0.47	0.47	1.88	128	51
		(0.23-0.47)	(0.47-1.88)		(0.94-1.88)		
LBT-3	30	0.47	0.23	0.47	1.88	64	28
			(0.23-0.47)		(1.88-3.75)		
SAAP-148	0.47	0.94	0.94	0.47	1.88	0.7	0.3
			(0.94-1.88)	(0.23-0.94)	(0.47-3.75)		
LL-37	>60	1.88	60	>120​	>120​		
		(0.94-1.88)					

^a^ Hemolytic concentration (HC) - Maximum peptide concentration that causes more than 30% lysis of human erythrocytes after 45 min of peptide exposure.

^b^ Bactericidal activity is expressed as the lethal concentration 99.9% (LC99.9 _(µM)_), the lowest peptide concentration (µM) that killed ≥99.9% of bacteria after 18 h of incubation. Results are medians (with ranges) derived from three independent experiments. Where no range is specified, the LC99.9 value remained consistent.

^c^ Selectivity index - HC/Gm of Lethal Concentration 99.9% (LC99.9). A larger value indicates higher specificity towards prokaryotic cells over eukaryotic cells.

^d^ Gm- Geometric mean of LC99.9 values from the *Staphylococcus aureus* and *Acinetobacter baumannii*.

Both LBT and LBT-1 exhibited low hemolytic activity, with <30% hemolysis at the highest concentration tested (60 µM), resulting in high selectivity indices. In contrast, LBT-2 induced increased hemolysis, reaching the 30% threshold at 60 µM. LBT-3, which incorporates a D-stereoisomer core, showed the highest hemolytic activity among all variants, exceeding 30% hemolysis at 30 µM. Among the reference peptides, SAAP-148 displayed strong antimicrobial activity but was also strongly hemolytic (30% hemolysis at 0.47 µM). LL-37, while non-hemolytic at highest concentration tested, showed poor antimicrobial activity particularly in the presence of plasma (LC99.9 > 120 µM for *A. baumannii*). Hence, the lead candidate LBT-1 was selected and parent peptide LBT was taken for further functional characterization based on the selectivity index, taking into account: (i) antimicrobial activity against both bacterial species in RPMI, (ii) retention of activity in 50% plasma, and (iii) minimal hemolytic activity.

### 2.2 LBT-1 exhibits potent bactericidal activity against ESKAPE(E) pathogens

The bactericidal activity of LBT and LBT-1 was evaluated against MDR strains of the ESKAPE(E) panel (Table 2), along with SAAP-148 as reference peptide. Both peptides showed higher activity against Gram-positive than against Gram-negative bacteria. LBT-1 demonstrated strong bactericidal activity against *Enterococcus faecium* and *S. aureus* in both RPMI and 50% plasma. Against *S. aureus*, LBT-1 showed superior potency in plasma (<0.12 µM), outperforming LBT (0.48 µM) and SAAP-148 (0.94 µM). In contrast, activity against Gram-negative *Klebsiella pneumoniae* and *P. aeruginosa* was reduced, especially in plasma for both peptides. Against *P. aeruginosa*, the LC99.9 values increased 16-fold (1.88–30 µM) for LBT and 4-fold (7.5–30) for LBT-1 in the presence of plasma. Interestingly, both peptides showed enhanced activity against *Enterobacter cloacae* in 50% plasma, with LC99.9 values decreasing by 32-fold, an effect not observed with SAAP-148. Against colistin-resistant *Escherichia coli*, both LBT and LBT-1 retained potent activity (LC99.9: 0.94–1.88 µM). Plasma testing was not performed for *E. coli* due to known complement sensitivity. Overall, LBT-1 exhibited potent activity against Gram-positive pathogens and colistin-resistant *E. coli*, with reduced activity against Gram-negative bacteria in plasma, while showing a unique plasma-enhanced effect against *E. cloacae.* These findings indicate plasma influences peptide activity in a species-dependent manner.

**TABLE 2:** Bactericidal activity (LC99.9 (µM)) of LBT, LBT-1 and the reference peptide SAAP-148 against MDR strains, part of the ESKAPE(E) panel. Results are presented as median (with ranges) from three independent experiments. Peptides were tested in a two-fold serial dilution series of 120–0.12 µM. Fungicidal activity of the peptides were tested against *C. auris* and *C. albicans* in a two-fold serial dilution series of 30–0.12 µM. Results are from two independent experiments. In case no range is specified, the LC99.9 value was identical in both experiments.

Species		LBT		LBT-1		SAAP-148	
		RPMI	50% plasma	RPMI	50% plasma	RPMI	50% plasma
*E. faecium*	LUH 15122	1.88	0.94	0.94	0.94	0.94	0.94
		(1.88-0.94)	(0.94-0.48)		(0.94-0.48)	(0.94-0.48)	(1.88-0.94)
*S. aureus*	LUH 14616	0.94	0.48	0.94	<0.12	0.94	0.94
		(0.94-0.48)	(0.48-0.23)	(0.94-0.48)		(0.94-0.48)	(1.88-0.94)
*K. Pneumoniae*	LUH 15104	7.5	>30	3.75	>30	1.875	7.5
							(7.5-3.75)
*A. baumannii*	RUH 875	0.47	1.88	0.94	1.88	0.47	0.94
					(0.94-1.88)		
*P. aeruginosa*	LUH 15103	1.88	30	7.5	30	3.75	15
		(1.88-0.94)		(7.5-3.75)			(30-15)
*E. cloacae*	LUH 15114	7.5	0.48	3.75	0.12	3.75	3.75
		(7.5-3.75)		(3.75-1.88)			
*E. coli*	LUH 15117	1.88	—	0.94	—	0.94	—
*C. albicans*	SC5314	3.75–7.5	30–60	1.88–3.75	15	15–60	≥120
*C. auris*	111 (clade I)	7.5	60	15	120	60–120	≥120

“—“Indicates not determined.

### 2.3 LBT-1 exhibit potent activity against *Candida albicans* and MDR *Candidozyma auris*

The antifungal activity of LBT and LBT-1 was evaluated in comparison to SAAP-148 (Table 2). In RPMI, both LBT peptides showed strong activity against *C. albicans* SC5314 (LC99.9: 1.88–7.5 µM) and *C. auris* 111 (LC99.9: 7.5–15 µM), whereas SAAP-148 required higher concentrations to achieve similar killing of *C. albicans* (15–60 µM) and *C. auris* 111 (60–120 µM). The presence of 50% plasma reduced the activity of all three peptides, with a more pronounced decrease observed against *C. auris*. Nevertheless, both LBT and LBT-1 retained potent fungicidal activity under plasma conditions, demonstrating superior potency over SAAP-148 and consistent activity across both fungal species.

### 2.4 LBT-1 exhibits improved and more rapid bactericidal activity compared to LBT peptide

**FIGURE 1:**
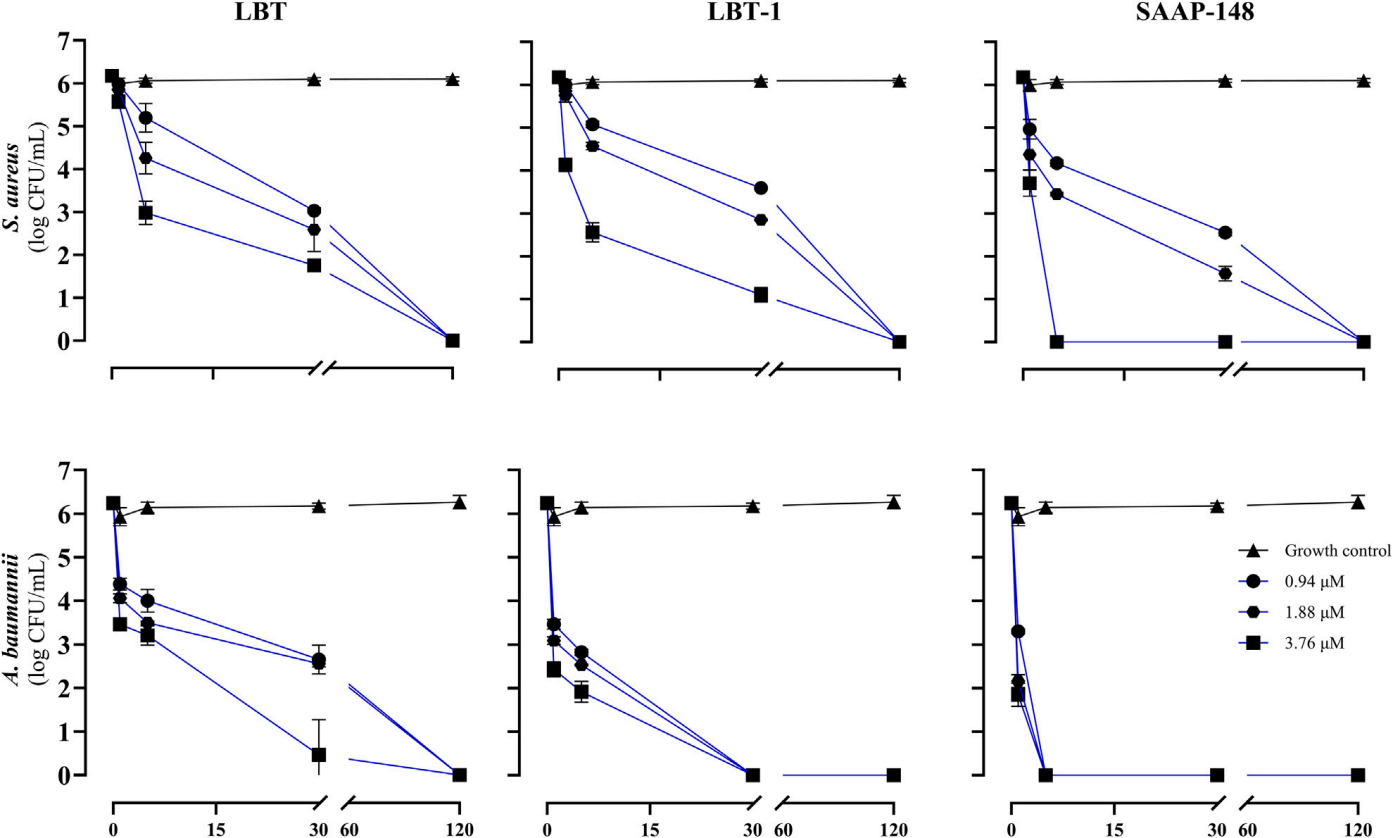
Bactericidal kinetics of LBT, LBT-1 and reference SAAP-148 peptide. **(A)** Against *Staphylococcus aureus* JAR060131 and *Acinetobacter baumannii* RUH875 **(B)** in RPMI. Peptides concentrations are indicated by symbols: circle = 0.94 µM, pentagon = 1.88 µM, and square = 3.75 µM. Bacterial survival (CFU/mL) was measured at 1, 5, 30, and 120 min post treatment. Growth control without peptide (triangle) was included for reference. Data represents the mean ± SD from each condition performed in triplicates.

The bactericidal kinetics of LBT and LBT-1 were evaluated against *S. aureus* and *A. baumannii* and compared to SAAP-148, known for its rapid and potent microbicidal activity. SAAP-148 achieved complete killing of *S. aureus* JAR060131 at 3.75 µM within 5 min (Figure 1A) and a >3-log reduction in *A. baumannii* RUH875 within 1 min at 3.75 µM (Figure 1B). In comparison, both LBT and LBT-1 exhibited slower killing kinetics against *S. aureus* and *A. baumannii* and >3-log reduction in *S. aureus* was observed within 5 min at 3.75 µM for both peptides and, whereas complete bacterial eradication required up to 120 min for all tested concentrations. Complete killing was confirmed by plating treated bacterial suspensions on BA plates. Interestingly, at 3.75 µM, LBT-1 achieved a >3-log reduction within 1 min and complete killing at within 30 min, whereas LBT required 120 min at the same concentrations, indicating improved activity of LBT-1 against *A. baumannii*.

### 2.5 LBT-1 and LL-37 display similar cytotoxicity to human skin cells

The cytotoxic concentration of LBT, LBT-1, SAAP-148, and LL-37 was determined using metabolic activity (WST-1) and membrane permeabilization (LDH) assays in human keratinocytes and dermal fibroblasts (Table 3). A peptide was considered cytotoxic when it reduced cell metabolic activity below 70% and induced more than 30% membrane permeabilization. Within the defined cytotoxicity threshold (section 4.5.2), LBT-1 and LL-37 exhibited similar tolerance at 15 µM in keratinocytes and 7.5–15 µM in fibroblasts. SAAP-148 displayed the lowest cytotoxic thresholds, at 1.88–3.75 µM. Across all peptides tested, fibroblasts were more sensitive than keratinocytes, particularly in terms of membrane permeabilization. Dose dependent cytotoxic effects of LBT and LBT-1 peptides on these cells are shown in Supplementary Figure S1. Hence, concentrations of <30 µM for LBT and <15 µM for LBT-1 were identified as safe and used for subsequent cell-based assays.

**TABLE 3:** Cytotoxic profiles of LBT and LBT-1 peptides tested in human skin fibroblasts and human epidermal keratinocytes. Values presented are cytotoxic Concentrations (μM) of LBT, LBT-1, and reference peptides-SAAP-148 and LL-37 after 24h of exposure. Peptides were tested in a two-fold serial dilution range (60–0.12 µM). Cytotoxic range was defined as the concentration causing <70% metabolic activity measured by WST and >30% membrane permeation as measured by LDH leakage.

Peptide	Cytotoxicity (µM)			
	Human keratinocytes		Human dermal fibroblasts	
	LDH leakage	Metabolic activity	LDH leakage	Metabolic activity
LBT	30	30	15	30
LBT-1	15	15	7.5	15
SAAP-148	3.75	3.75	1.88	3.75
LL-37	15	15	7.5	15

### 2.6 LBT-1 neutralizes endotoxin-induced pro-inflammatory cytokine production

**FIGURE 2:**
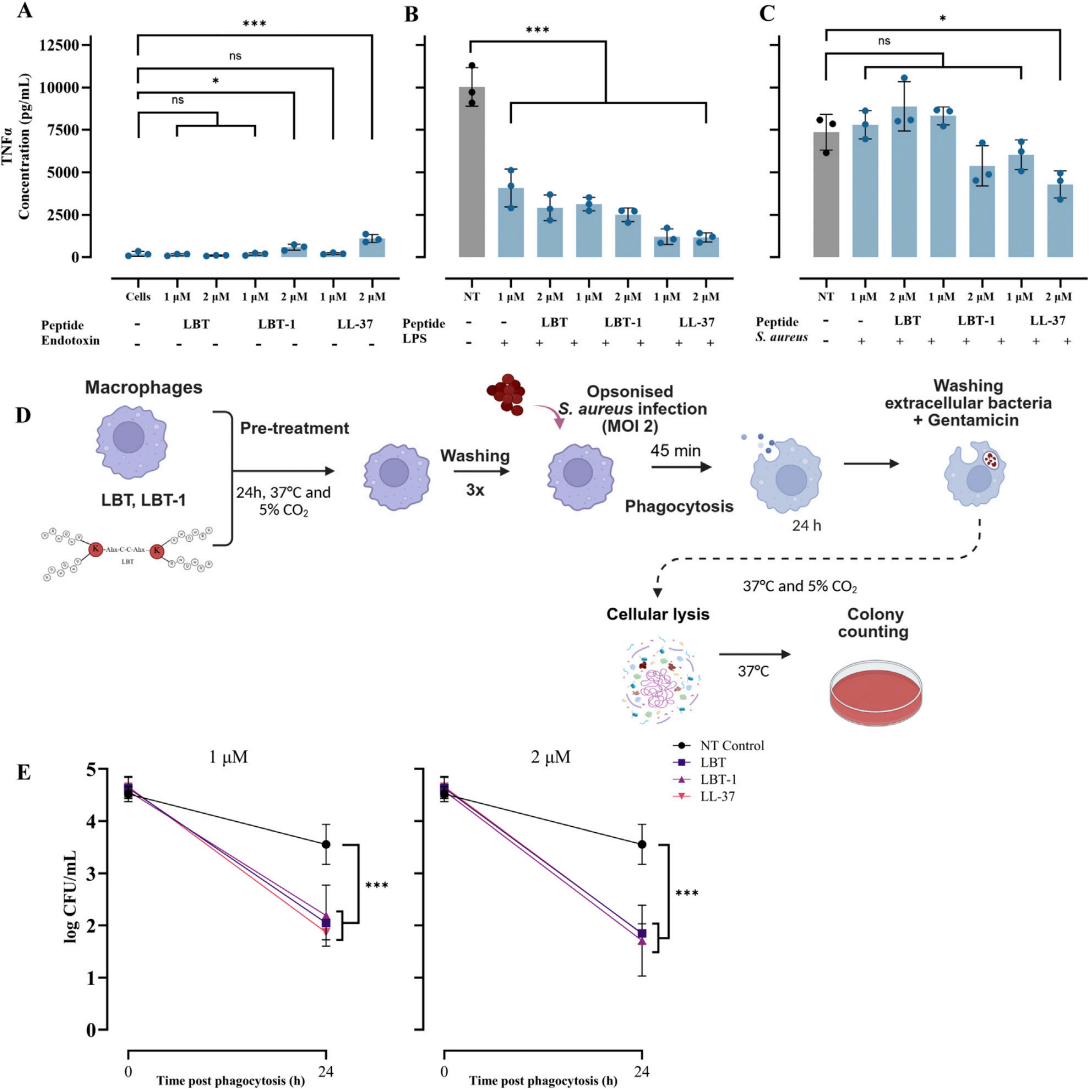
LBT peptides attenuate endotoxin induced pro-inflammatory cytokine induction in macrophages and enhance bacterial clearance in macrophages. **(A)** Macrophages treated with only peptides without endotoxin stimulation, following 4 h peptide treatment, the levels of TNFα in the supernatants was determined using ELISA and LL-37 served as positive control. Cells represent unstimulated macrophages without LPS or bacterial challenge, while NT (no treatment) indicates stimulated macrophages without peptide treatment. **(B)** Measured TNFα levels in macrophages stimulated with LPS and treated with peptides. **(C)** Measured TNFα levels in macrophages stimulated with UV-inactivated *Staphylococcus aureus* and treated with peptides. Statistical significance was assessed using one-way ANOVA, followed by Tukey’s *post hoc* test and compared to unstimulated cells control **(A)** or compared to NT control **(B,C)**. Schematic overview of experimental set-up intracellular killing assay in macrophages infected with *Staphylococcus aureus* at a MOI of 2 is shown **(D)**. Pre-treatment with LBT, LBT-1 or LL-37 at 1 μM[(E) left] and 2 μM [(E) right] significantly enhanced the ability of macrophages to reduce intracellular *Staphylococcus aureus* burdens 24 h post-phagocytosis. Bacterial survival was quantified by enumerating CFU following macrophage lysis and plating. Data are presented as mean ± SD of 3 biological replicates. Here, statistical significance was assessed using one-way ANOVA, followed by Tukey’s *post hoc* test and compared to no treatment control.

To assess the ability of LBT and LBT-1 to neutralize bacterial endotoxins, human macrophages were stimulated with either LPS, as a gram-negative stimuli, or UV- inactivated *S. aureus*, representing gram positive stimuli, in the presence or absence of peptides. A schematic overview of the experimental workflow is provided in Supplementary Figure S2A. LL-37 served as the positive control and following stimulation and treatment, levels of the pro-inflammatory cytokine TNFɑ were quantified. LPS stimulation of the macrophages resulted in a robust TNFɑ response (10,030 pg/mL; *p* < 0.001), and macrophages treated with peptides alone (LBT-1 and LL-37 at 2 µM) induced minimal but significant TNFɑ induction compared to unstimulated cells (Figure 2A). Co-treatment with LPS and peptides significantly reduced TNFɑ levels in a concentration-dependent manner (*p* < 0.001 vs. NT, for all peptides; Figure 2B), indicating an endotoxin-neutralizing effect similar to that of LL-37.

When macrophages were stimulated with UV-inactivated *S. aureus*, co-treatment with peptides did not significantly reduce TNFɑ production, except for LL-37 at 2 µM (*p* < 0.05; Figure 2C), suggesting limited neutralization. Overall, these results demonstrate that both LBT and LBT-1 effectively neutralize LPS-induced pro-inflammatory responses in human macrophages.

### 2.7 LBT-1 pre-treatment enhances intracellular bacterial clearance in macrophages

Here we investigated whether LBT or LBT-1 pre-treatment enhances macrophage-mediated intracellular killing of *S. aureus* RN4220. LL-37, known to promote intracellular bacterial killing (Tang et al., 2015) was included as a positive control. A schematic overview of the experimental workflow is provided in Figure 2D. Peptide pre-treatment at 1 μM and 2 µM did not significantly affect bacterial uptake, as determined by CFU counts from macrophages lysed 45 min after initiation of phagocytosis (defined as t = 0). However, at 24 h post-phagocytosis, macrophages in the untreated control group showed minimal reduction in intracellular bacterial load, whereas pre-treatment with LBT or LBT-1 led to a significant reduction. Both LBT and LBT-1 at 1 µM significantly enhanced intracellular bacterial clearance (*p* < 0.001 for all peptides), achieving levels comparable to LL-37 (Figure 2E, left). Increasing the peptide concentration to 2 µM did not result in further reductions in CFU counts, (*p* < 0.001 for all peptides; Figure 2D, right).

### 2.8 LBT and LBT-1 enhance keratinocyte migration post-wounding in a scratch wound assay

**FIGURE 3:**
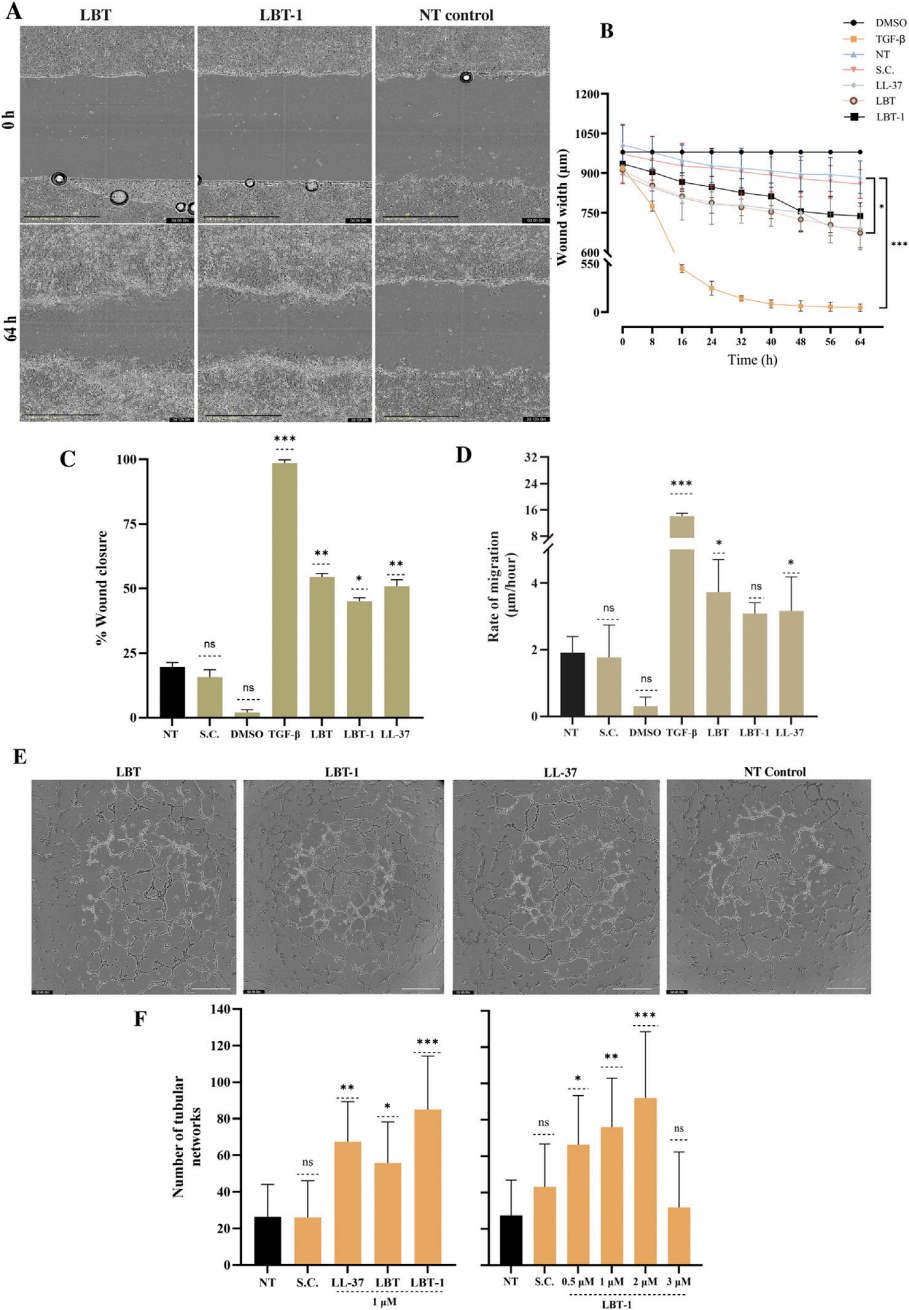
Evaluation of *in vitro* wound healing and angiogenic potential of LBT and LBT-1 peptide. **(A)** Representative phase-contrast images of human keratinocytes scratch wounds at 0 h and 64 h after treatment with 1 μM LBT, LBT-1, or the no treatment control. Scale bar = 400 µm. **(B)** Quantification of wound width over 64 h, based on images captured every 8 h, with DMSO as negative control, TGF-β as positive control, and LL-37 as positive peptide control. **(C)** %Wound closure at 64 h, calculated relative to initial wound area at 0 h. **(D)** Rate of cell migration (µm/h) calculated from wound width at 0 h and 64 h over the total time of the assay (64 h). Data are presented as mean ± SD from triplicates and compared to no treatment (NT). **(E)** Representative images of capillary-like tubule formation by differentiated human endothelial cells after 4 h treatment with peptide or no treatment. Scale bar = 800 µm. [**(F)**, left] Quantification of tubular networks formed following treatment with LBT, LBT-1, LL-37, tested at 1 μM, no treatment (NT), and solvent control (SC). [**(F)**, right] Dose-dependent effect of LBT-1 (0.5–3 µM) on tubule formation. The initial peptide screening shown in panel F (left) and the concentration-dependent analysis in panel F (right) were performed as separate experiments. Statistical significance was assessed using one-way ANOVA, followed by Tukey’s *post hoc* test and compared to no treatment control. Data are presented as mean ± SD from at least three independent experiments.

The ability of the peptides to promote cell migration post wounding was assessed using a 2D *in vitro* scratch wound assay. LL-37, known for its wound healing properties (Carretero et al., 2008), served as a reference peptide. Positive control, TGF-β treatment resulted in near-complete wound closure at 60 h post-injury, whereas DMSO-treated cells showed minimal migration, validating the assay. Representative images from the scratch assay starting at 0 h and after 60 h treatment with LBT and LBT-1, tested at 1 µM and no treatment control are shown in Figure 3A. TGF-β resulted in highest wound width reduction (*p* < 0.001) and among peptides, only LBT significantly reduced wound width (*p* < 0.05; Figure 3B) compared to the untreated control, demonstrating a strong pro-migratory effect on the wounded cells. This was further supported by the percentage of wound closure over time (Figure 3C), where TGF-β (*p* < 0.001) resulted in highest activity. LBT (*p* < 0.01), LBT-1 (*p* < 0.05) and LL-37 (*p* < 0.01) treatment led to significant wound closure compared to the no treatment control. A similar trend was observed in the rate of cell migration (Figure 3D), with TGF-β treatment (*p* < 0.001) resulting in faster wound closure and among peptides, LBT treatment resulted in the highest migration (*p* < 0.05), followed by LL-37 (*p* < 0.05), and LBT-1 (ns) compared to no treatment. LBT significantly enhanced cell migration resulting in wound closure, showing the strongest pro-migratory effect among the tested peptides, though lower than the positive control TGF-β.

### 2.9 LBT-1 demonstrates superior enhancement of tubular network

To evaluate the angiogenic potential of the peptides in human endothelial cells, tube formation was quantified, using LL-37 as a positive control. Representative images of the tubular structures are shown in Figure 3E. LBT-1 treatment (at 1 µM) resulted in dense and well-connected tubular networks, significantly increasing the number of networks compared to the untreated control (*p* < 0.001). Notably, LBT-1 surpassed the effect of the positive control LL-37 (*p* < 0.01; Figure 3F, left). LBT (*p* < 0.05) also enhanced tube formation, though the resulting networks were less extensive and less interconnected than those observed with LBT-1. To further study dose dependency, a concentration series of LBT-1 was tested (Figure 3F, right). Tube formation peaked at 2 µM (*p* < 0.001), with lower concentrations (0.5 and 1 µM) still resulting in significant increase in tubular structure formation (*p* < 0.05 and *p* < 0.01, respectively) and at 3 μM, tube formation declined.

### 2.10 LBT-1 demonstrates improved bactericidal activity against MRSA in an *ex vivo* human skin infection model

**FIGURE 4:**
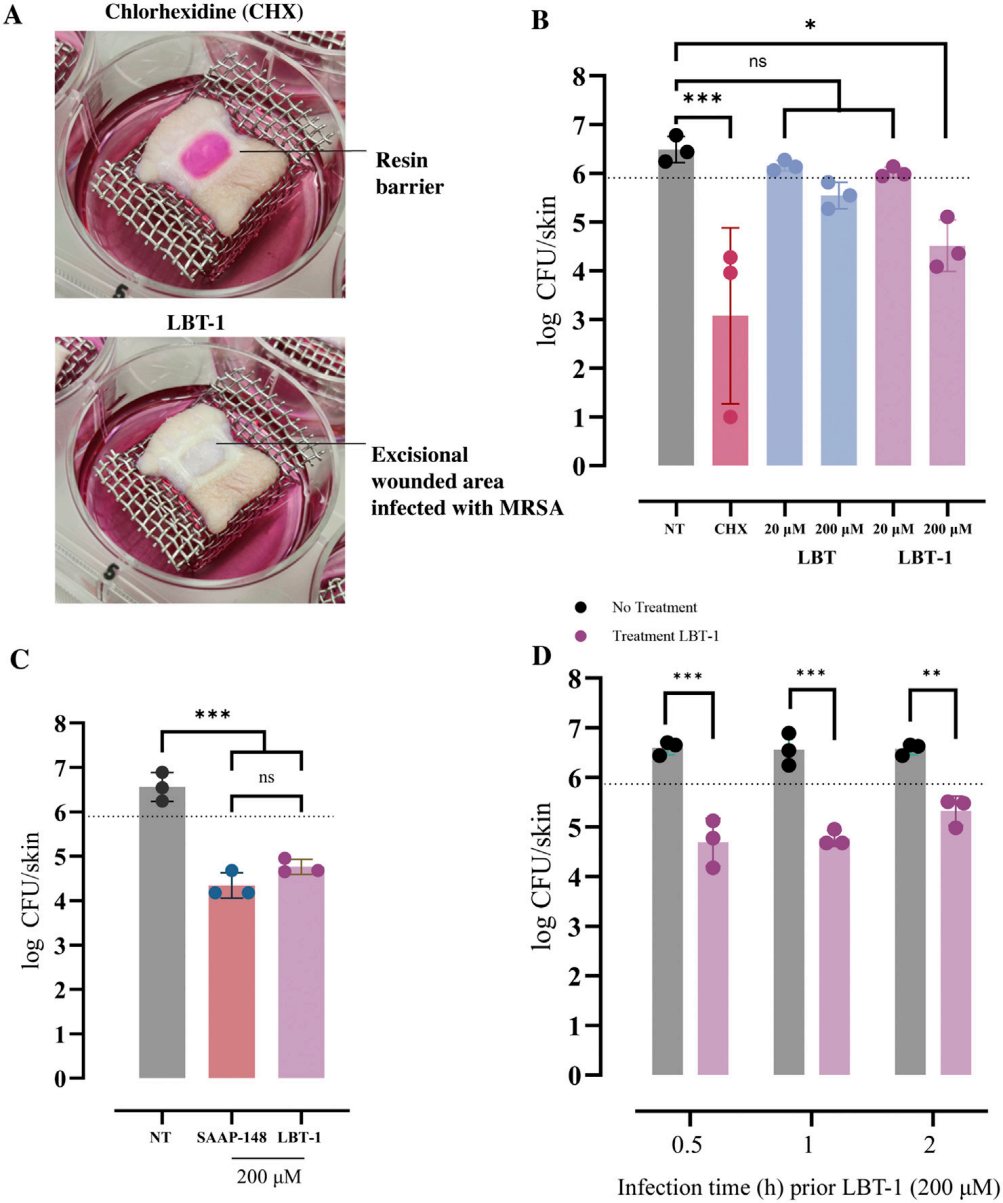
Bactericidal activity of LBT and LBT-1 peptides in an *ex vivo* human skin infection model infected with MRSA LUH14616. **(A)** Images of infected 1 cm^2^ wounded human skin grafts following treatment with chlorhexidine (CHX; top) and LBT-1 (bottom) at 200 µM. **(B)** Quantification of bacterial burden (log CFU/skin) following infection with MRSA and treatment with LBT and LBT-1 at 20 μM and 200 µM. PBS is the no treatment control, while CHX is positive control. The dotted line indicates the bacterial inoculum used for infection. **(C)** Comparison of bacterial loads in infected skin treated with 200 µM LBT-1 or SAAP-148, compared to the untreated control (NT). **(D)** Influence of infection duration (0.5 h, 1 h, and 2 h post-infection) prior to treatment with LBT-1 (200 µM) on CFU reduction. Statistical significance was assessed using one-way or two-way ANOVA, followed by Tukey’s *post hoc* were performed for comparison between log CFU counts of treatment and no treatment groups or Sidak’s test for multiple comparisons, between different time points before initiation of peptide treatment. Data are presented as mean ± SD from triplicate skin samples per condition.

An *ex vivo* human skin infection model was employed to evaluate the bactericidal activity of LBT and LBT-1 against *S. aureus* LUH14616 (MRSA). In this model, wounded skin samples were maintained at the air-liquid interface and topically infected with MRSA (1 × 10^5^ CFU/graft), followed by peptide treatment. Chlorhexidine and PBS served as positive and negative controls, respectively. Following treatment, tissues were homogenized, and bacterial counts were determined by CFU enumeration. Representative images of the infection model are shown in Figure 4A. Both peptides had previously demonstrated potent *in vitro* activity against this bacteria (LC99.9 < 1 μM; Table 2). After 4 h of LBT and LBT-1 treatment, bacterial load was quantified and compared to untreated control (PBS) (Figure 4B). In the untreated control, bacterial levels slightly exceeded the initial inoculum, confirming growth and persistence of *S. aureus*. Chlorhexidine treatment significantly reduced bacterial burden (*p* < 0.001). LBT treatment at both 20 μM and 200 µM led to minimal and non-significant reductions in CFU counts compared to the untreated control. In contrast, LBT-1 induced a dose-dependent reduction in bacterial load, with a significant decrease observed at 200 µM (*p* < 0.05), highlighting its enhanced antimicrobial activity over the LBT. To further compare activity, LBT-1 and SAAP-148 were both tested at 200 µM. After 4 h of treatment, both peptides significantly reduced bacterial load compared to the untreated control (*p* < 0.001 for both peptides; Figure 4C). The difference in activity between LBT-1 and SAAP-148 was not statistically significant. Finally, to evaluate the influence of infection duration prior to treatment, skin samples were infected with *S. aureus*, and left untreated for 0.5, 1, or 2 h before initiating treatment with LBT-1 (200 µM) for 4 h. LBT-1 significantly reduced bacterial load in all groups (*p* < 0.001 for 0.5 and 1 h; *p* < 0.01 for 2 h; Figure 4D). No significant differences were observed between the 0.5 h and 1 h infection groups, suggesting that the bacteria remain accessible and susceptible to LBT-1 within this time window. However, when treatment was initiated 2 h post-infection, the reduction in bacterial load was slightly lower, potentially reflecting decreased accessibility or susceptibility within the tissue environment.

## 3 Discussion

### 3.1 Structural modifications and the impact on selectivity

This study focused on enhancing the therapeutic potential of the LfcinB tetramer (LBT) for chronic wound treatment by introducing modifications at the branching core. By substituting the two lysine residues with non-natural lysine derivatives, we investigated the effect of changes in motif orientation and linker flexibility on peptide functionality. Among the three novel tetrameric variants, LBT-1 (DAP) emerged as the lead candidate. Although other variants maintained exceptional antimicrobial potency against *S. aureus* and *A. baumannii*, their modifications increased hemolytic activity slightly or moderately (LBT-2, LBT-3, respectively) compared to the parent LBT.

In case of LBT-1, the side chain of DAP may have contributed to this favorable characteristic as in α-helical AMPs DAP reduced hydrophobicity and limited nonspecific membrane interactions (Mant et al., 2019). Similar effects have been reported in dendrimer peptide architectures, where shorter amino-terminal side chains such as in DAP were proposed to alter spatial charge presentation and reduce non-specific interactions with mammalian membranes (Stach et al., 2012). In LBT-2 and LBT-3, the introduction of additional hydrophobicity or altered branching core geometry respectively, could have likely increased the probability of peptide insertion into zwitterionic mammalian membranes, thereby explaining the increase in hemolytic activity.

Importantly, LBT-1 demonstrated broad-spectrum activity against clinically relevant MDR pathogens within the ESKAPE(E) group, which are major contributors to healthcare associated and chronic wound infections. Due to increasing prevalence, and resistance to conventional antibiotics, the World Health Organization (WHO) has classified these as high-priority pathogens requiring urgent development of new therapeutics (WHO, 2017). In particular, MRSA remains one of the leading causes of hospital and community acquired infections worldwide, accounting for a substantial proportion of the global antimicrobial resistance (AMR) burden.

Notably, LBT-1 not only retained but even showed enhanced bactericidal activity in the presence of plasma, particularly against MRSA, *E. faecium*, and *E. cloacae,* suggesting improved activity in physiological conditions. This enhanced activity of peptides against Gram-positive bacteria may arise from potential plasma proteins or complement-mediated synergy. Similar observations have been reported for other antimicrobial peptides, where plasma enhanced activity against certain bacterial species (Citterio et al., 2016). Interestingly, plasma-dependent enhancement in bactericidal activity was only observed for *E. cloacae* among the Gram-negative species tested, and not for *K. pneumoniae*, *A. baumannii,* or *P. aeruginosa* (Heesterbeek et al., 2019). This may be due to the differential protective role of the outer membrane, variations in LPS composition, and reduced complement susceptibility, which may limit peptide access or penetration and prevent synergistic effects in these bacteria (Smart et al., 2017; Simpson and Trent 2019). Importantly, the plasma testing performed in this study provides a physiologically relevant assessment of peptide bactericidal activity. These findings highlight that plasma-dependent modulation of peptide activity should be considered when translating *in vitro* potency to *in vivo* or clinical applications, and that in some cases, effective doses may be lower than those determined by MIC assays in laboratory media. Further, LBT-1 also showed rapid and improved bactericidal kinetics against *S. aureus* and *A. baumannii*, achieving faster killing compared to the parent LBT peptide. Additional to bactericidal activity, LBT-1 showed potent antifungal activity against *C*. *albicans* and the emerging MDR fungal pathogen *C*. *auris*. Together, these findings highlight the broad-spectrum and potent activity of LBT-1, aligning with WHO priorities for the development of novel therapeutics targeting these MDR pathogens.

### 3.2 Anti-inflammatory activity of the tetramers

Chronic wound inflammation often persists despite bacterial clearance, driven by residual bacterial remains as immune triggers such as endotoxins and other cellular debris (Krzyszczyk et al., 2018). Most antibiotics fail to resolve this prolonged immune activation, highlighting the need for therapeutics that also modulate inflammation (Wolf et al., 2017). Here, both the LBT-1 and LBT peptide significantly reduced TNFɑ levels secreted by macrophages stimulated with purified LPS from *E.coli*, indicating their ability to neutralize endotoxin and dampen inflammatory signaling. The observed effect likely results from direct peptide–LPS interaction, possibly through electrostatic binding, preventing activation of the CD14/TLR4 signaling pathway in macrophages (Appelmelk et al., 1994). However, this anti-inflammatory effect was not observed when macrophages were stimulated with UV-inactivated *S. aureus*. Although cationic peptides have been reported to neutralize lipoteichoic acid (LTA)-mediated cytokine production (Brunetti et al., 2020), *S. aureus* intact cells present a more complex inflammatory stimulus than purified LPS or LTA alone. Their cell envelope contains multiple Toll-like receptor (TLR) agonists, including peptidoglycan (PG), LTA, lipoproteins, and other pathogen-associated molecular patterns (PAMPs), many of which may be embedded within the membrane (Ferraro et al., 2021). This complexity likely limits peptide accessibility and binding, resulting in sustained TNFɑ production despite peptide treatment. Interestingly, when macrophages were treated with peptides alone, without endotoxin stimulation, only LBT-1 and the positive control LL-37 at 2 µM induced a slight but significant TNFɑ induction compared to unstimulated cells. This likely reflects macrophage activation by these cationic peptides (Kahlenberg and Kaplan, 2013).

### 3.3 Peptide mediated intracellular bacterial killing

Effective chronic wound resolution also requires clearance of intracellular bacterial reservoirs. *Staphylococcus aureus* can persist inside macrophages, evading immune surveillance and delaying healing (Pidwill et al., 2021). In our study, pre-treatment of macrophages with LBT or LBT-1 significantly reduced intracellular *S. aureus* load, suggesting that these peptides may enhance antimicrobial activity of macrophages. While the precise mechanism remains unclear, this effect may be linked to immunomodulatory actions on phagosome maturation or intracellular signaling pathways, as previously described for other lactoferricin-derived peptides (Silva et al., 2017). However, it cannot be excluded that peptide pre-treatment may have directly or indirectly facilitated gentamicin uptake or altered macrophage membrane properties, thereby increasing permeability and contributing to the enhanced bactericidal activity compared to untreated macrophages. Nevertheless, by dampening excessive inflammatory responses while simultaneously boosting macrophage bactericidal activity, these tetrameric peptides could contribute to a balanced immune response that efficiently clears intracellular *S. aureus* without excessive host cell damage.

### 3.4 Tissue repair

Keratinocyte migration is a fundamental process in wound re-epithelialization and restoration of tissue integrity and represents a critical final step in chronic wound healing. Cell migration is tightly coordinated by a network of signaling pathways involving growth factors, cytokines, immune mediators, and matrix interactions (Ramirez et al., 2014). In our study, the observed enhancement of keratinocyte migration and wound closure following treatment with the tetrameric peptides LBT and LBT-1, highlights their potential role in epithelial repair mechanisms. Beyond re-epithelialization, angiogenesis plays a pivotal role in restoring tissue function by restoring supply of nutrients, oxygen, and immune cells to the wound bed. Using an established basement membrane matrix model for the initial steps of angiogenesis (Irvin et al., 2014), LBT-1 was found to significantly promote endothelial tube formation, surpassing the angiogenic effect of LL-37 and the parent LBT peptide. This response was both robust and dose dependent. While the precise mechanism remains to be elucidated, the host-defense nature of this tetramer suggests that it could engage cell-surface receptors or modulate signaling pathways involved in endothelial migration and tubulogenesis. These findings show that incorporation of DAP at the branching core could enhance angiogenic properties of these multifunctional AMPs.

### 3.5 *Ex vivo* human wounded skin infection model

The *ex vivo* human wounded skin infection model not only preserves the anatomical architecture of human skin but also allows reproducible activity assessment of antimicrobial agents under conditions that closely mimic clinical wound infections. In our study, this model was adapted by the creation of a polymerized resin barrier, which allowed for confined addition of bacterial inoculum and reproducible assessment of localized treatment to the wounded area. Upon careful selection of the resin, we employed OpalDam, a light-cured resin commonly used in dentistry (Becerra et al., 2025). Its passive adhesion and biocompatibility made it suitable for confining the wound area without affecting tissue viability. Hence, this model offers a reproducible and physiologically relevant platform that is highly suitable for preclinical testing of topical antimicrobials. Within this model, the DAP-modified tetrameric peptide, LBT-1, demonstrated improved antimicrobial activity compared to the parent, LBT peptide, leading to significant reduction in MRSA burden. While LBT failed to significantly reduce bacterial load at either concentration tested, LBT-1 showed antimicrobial activity, highlighting the impact of the DAP substitution on peptide potency in this clinically relevant human tissue environment. Notably, the effective bactericidal concentration of LBT-1 (200 µM) was ∼200-fold higher than its LC99.9, likely reflecting reduced bacterial accessibility or reduced peptide diffusion in complex tissue environment. This finding highlights that higher concentrations are often required to achieve comparable *in vitro* antimicrobial effects in *ex vivo* or *in vivo* settings. Furthermore, LBT-1 remained effective with longer periods of infection prior to treatment, showing significant activity even when applied after 2 h of MRSA colonization.

## 4 Conclusion

This study identifies the DAP-modified tetrameric peptide LBT-1 as a promising multifunctional candidate for chronic wound management. LBT-1 combines potent antimicrobial activity, immune modulation, and tissue-regenerative effects, targeting three core pathological features of chronic wounds and is superior to the parent peptide LBT. Evaluation of the parent LBT peptide also revealed previously unknown beneficial characteristics of this peptide and provided an essential benchmark for understanding the functional impact of branching core modifications. The enhanced angiogenic activity *in vitro* and importantly the antimicrobial activity observed in the *ex vivo* model represents a key step towards *in vivo* validation, providing evidence that LBT-1 retains potency in a physiologically relevant tissue environment. Together, these findings validate the peptide design strategy and support the continued development of LBT-1 as multifunctional peptide wound therapeutic. Future work will focus on optimizing delivery systems, evaluating long-term efficacy and safety in *in vivo* wound models, and advancing toward clinical translation.

## 5 Materials and methods

### 5.1 Peptide synthesis and purification

**FIGURE 5:**
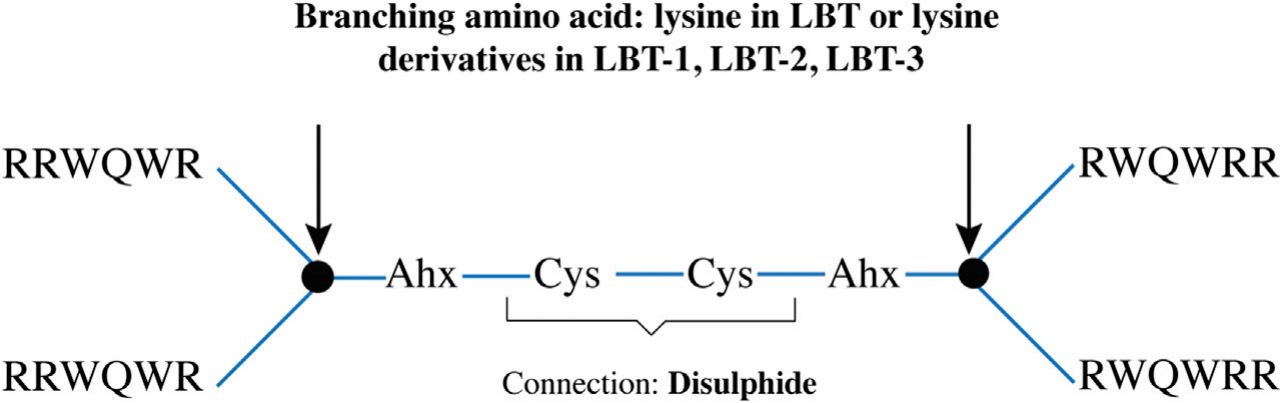
Schematic representation of the tetrameric peptide backbone, indicating branching residues (arrows) linked by two aminohexanoic acid (Ahx) spacer and cysteine disulphide bridge.

All the LBT peptides were synthesized on Tetagel-AM resin (Rapp Polymere) as previously described (Hiemstra et al., 1997), performing five parallel syntheses per peptide on a 10 µmol scale. Incorporation of Fmoc-DAP-Fmoc and Fmoc-DAPOA-Fmoc followed the same procedure as natural amino acids. After chain assembly, peptides were cleaved from the resin and side-chain protecting groups removed with trifluoroacetic acid (TFA). Crude products were precipitated using ether/pentane and analyzed by analytical UPLC/MS. The crude batches displayed comparable purity and composition and hence were pooled. They were purified via preparative chromatography on an ÄKTA system (Cytiva) using a water-to-acetonitrile gradient, followed by lyophilization. The purified branched peptides were dissolved in 20 mL of 20 mM Tris buffer (pH 8.3) containing 2 mL DMSO and subjected to air oxidation for 72 h to form the disulfide-linked products, as shown in the schematics (Figure 5). The oxidized peptides were subsequently repurified by ÄKTA, aliquoted into 10 mg portions, and stored at −20 °C until use. Aliquoting was based on quantification of stock solutions at an optical density at 280 nm (calculated using the extinction coefficient for tryptophan, ε = 5600 M^-1^ cm^-1^ per residue).

For MALDI-TOF MS analysis, peptides were dissolved in 100 µL acetonitrile/Milli-Q water (1:1, *v/v*) containing 0.1% TFA. 1 μL of this was mixed with 1 µL of a 10 mg/mL of 4-ACH (4-acetyl-1-cyclohexene-1-carboxylic acid) in acetonitrile/Milli-Q water (1:1, v/v) containing 0.1% TFA. The mixture was spotted onto a polished steel MALDI plate (MTP384, Bruker Daltonics), air-dried for ∼15 min, and introduced into a Microflex MALDI-TOF mass spectrometer (Bruker) for structure confirmation (Table 4).

**TABLE 4:** Structural details of LBT (LfcinB Tetramer) and modified peptides with variations in branching residues.

LfcinB tetrameric peptides	Sequence	Branching core residue	Residues	MALDI-TOF (M/Z)	
				MH^+^_Theoretical_	MH^+^_Experimental_
1. LBT	((RRWQWR)_2_KAhxC)_2_	L-Lysine	30	4598.4	4603
2. LBT-1	((RRWQWR)_2_DAPAhxC)_2_	L-2,3-diaminopropionicacid	30	4514.4	4515.2
3. LBT-2	((RRWQWR)_2_DAPOAAhxC)_2_	(2-(1,3-bisamino)propan-2-yl)oxy)acetic acid	30	4602.4	4603.6
4. LBT-3	((RRWQWR)_2_DKAhxC)_2_	D-lysine (D-Isomer of lysine)	30	4598.4	4599.9

Synthetic Antimicrobial and Antibiofilm peptide (SAAP-148) (Breij et al., 2018), and LL-37 (Gudmundsson et al., 1996) were used as reference peptides and dissolved in PBS to obtain a 1 mM stock solution. The lyophilized lactoferricin tetrameric peptides were dissolved in 50% (*v/v*) acetonitrile in Milli-Q water to obtain 1 mM stock solutions. All stock solutions were stored at −20 °C until use.

### 5.2 Microorganisms and culture

Bacterial strains used were *S. aureus* JAR060131 (Campoccia et al., 2008), *S. aureus* RN4220 expressing mCherry fluorescent protein (Veneman et al., 2013) and multiple MDR clinical strains belonging to the ESKAPE(E) panel: *Enterococcus faecium* LUH15122, *S. aureus* LUH14616, *Klebsiella pneumoniae* LUH15104, *A. baumannii* RUH875, *Pseudomonas aeruginosa* LUH15103, *Enterobacter cloacae* LUH15114, and colistin-resistant *Escherichia coli* LUH15117 (Breij et al., 2018). The fungal strains used were *Candidozyma auris* (formerly known as *Candida auris* (Liu et al., 2024)) strain 111 and *Candida albicans* SC5314 (ATCC MYA-2876). Bacterial and fungal strains were revived from frozen stocks by culturing on sheep blood agar (BA) plates (BioMérieux) at 37 °C overnight. Bacterial and fungal cultures were prepared by incubating 1-3 colonies in tryptic soy broth (TSB; Oxoid) or Sabouraud Dextrose broth (Difco), respectively, at 37 °C with shaking at 120 rpm overnight. *Staphylococcus aureus* RN4220 mCherry was grown in TSB supplemented with 10 μg/mL chloramphenicol (Merck). The overnight cultures were sub-cultured in fresh TSB or SD for 3 h to mid-logarithmic growth phase, washed twice with PBS and resuspended in Roswell Park Memorial Institute (RPMI)-1640 medium supplemented with 20 mM Hepes and L-glutamine without sodium bicarbonate (Sigma-Aldrich; referred to as RPMI) to the desired inoculum concentration, based on the optical density at 620 nm (OD_620_). RPMI was selected as it supports bacterial and fungal growth without compromising the antimicrobial activity of peptides (Schwab et al., 1999).

For intracellular infection experiments, mCherry-expressing *S. aureus* RN4220 was prepared as previously described (Cuypers et al., 2024). UV-inactivated *S. aureus* JAR060131 were prepared by exposing the bacterial suspension, with an OD_620_ corresponding 1 × 10^8^ CFU/mL, to UV light for 30 min with periodic agitation to ensure uniform inactivation. Absence of bacterial growth on BA confirmed complete UV-inactivation of the treated suspension.

### 5.3 Bactericidal and fungicidal activity

Bactericidal activity was assessed as previously described (Breij et al., 2018). Briefly, mid-logarithmic phase bacterial cultures were diluted in RPMI to 1 × 10^7^ CFU/mL. For fungicidal assays, fungal cultures were prepared in RPMI supplemented with 1% glucose (Merck) and diluted to 2–5 × 10^5^ CFU/mL. For each condition, 10 µL of the suspension was added to 90 µL of peptide-containing RPMI or RPMI supplemented with 50% (v/v) pooled human plasma (hereafter referred to as 50% plasma; final bacterial concentration 1 × 10^6^ CFU/mL, final fungal concentration 2–5 × 10^4^ CFU/mL. Human plasma from four healthy volunteers (Sanquin, Netherlands) was pooled, and stored at −20 °C until use. Peptides were tested similarly for fungicidal activity, after 18 h of incubation at 37 °C with shaking (120 rpm) under humidified conditions, bacterial and fungal survival was determined by plating 10 µL aliquots onto BA plates. Assays were performed in duplicate in round-bottom polypropylene microtiter plates (Costar) and repeated independently three times. Untreated controls included bacteria exposed to RPMI alone or RPMI with 50% plasma. Bactericidal and fungicidal activity was expressed as the lethal concentration 99.9% (LC99.9), defined as the lowest peptide concentration causing ≥99.9% reduction in numbers of CFU compared to the initial inoculum.

### 5.4 Bactericidal kinetics

Time-kill kinetics were evaluated against *S. aureus* JAR060131 and *A. baumannii* RUH875. Mid-logarithmic phase bacterial cultures were diluted to a final concentration of 1 × 10^6^ CFU/mL in PBS. Bacteria were incubated with 0.5-, 2-, and 4-fold the LC99.9. Assays were conducted in polypropylene tubes (Micronics) with a total volume of 500 µL. Control samples consisted of bacteria incubated in PBS alone. At 1, 5, 30, and 120 min, 50 µL samples were collected and immediately added to 50 µL of 0.05% (v/v) sodium polyanethol sulfonate (SPS; Sigma-Aldrich) in PBS to neutralize residual peptide activity (Dankert et al., 1995). Viable bacterial counts were determined by plating 10 µL of serial 10-fold dilutions onto BA plates. Colonies were counted after 24 h of incubation at 37 °C. All conditions were performed in triplicate.

### 5.5 Cytotoxicity assessment

#### 5.5.1 Hemolytic activity

Whole blood was collected from healthy volunteers after obtaining informed consent, using Vacuette EDTA tubes (Greiner Bio-One). The study was approved by the Institutional Review Board of AMC-UvA (BACON 1.8; approved 26-10–2018). Collected blood was centrifuged at 1500 rpm for 15 min at 4 °C, plasma removed, and erythrocytes were washed once with PBS before resuspension to 1% (v/v) in PBS. For hemolytic assessment, 180 μL of the prepared erythrocyte suspension was mixed with 20 μL of serially diluted peptide solutions (ranging from 60 to 0.03 μM in PBS). Controls included a no-hemolysis blank (PBS alone) and a 100% hemolysis control (cells lysed by 1% Triton X-100 (Sigma-Aldrich) in PBS. Samples were incubated at 37 °C for 45 min and centrifuged at 2500 rpm for 10 min. A 100 μL aliquot of the supernatant was collected, and absorbance at 540 nm using a UV−vis spectrophotometer (Synergy H1, Biotek) was measured. A peptide was considered hemolytic if it induced >30% hemolysis relative to the 100% hemolysis control. Results were averaged from triplicate measurements using erythrocytes collected from a single donor.

#### 5.5.2 Cytotoxicity for human skin fibroblasts

Human skin fibroblast cells (BJ; CRL-2522; ATCC) and HaCaT human keratinocyte (CLS Cell Lines Service, Cat. No. 300493) were cultured in Dulbecco’s Modified Eagle Medium (DMEM; Gibco) supplemented with 10% (v/v) fetal bovine serum (FBS; Sigma-Aldrich) and 1% (v/v) penicillin/streptomycin (Gibco). Cells (5 × 10^5^ cells/mL) were seeded in 96-well plates (100 μL/well; Thermo Scientific) and allowed to adhere for 24 h. After adhesion, monolayers were exposed to two-fold serial dilutions of the tetrameric and reference peptides (final concentrations: 60–0.05 µM) in fresh DMEM containing 2% (v/v) FBS, and incubated for 24 h at 37 °C in a humidified 5% CO_2_ atmosphere. Cell viability was assessed using the water-soluble tetrazolium salt (WST-1; Roche) assay, and membrane integrity was measured using the lactate dehydrogenase (LDH) release assay (Abcam), following manufacturer instructions. Absorbance was measured using the Synergy H1 microplate reader (Biotek). Results are expressed as percentages relative to untreated control (for WST-1) and positive lysis control (for LDH) and all experiments were performed in triplicate.

To evaluate the selectivity of peptides for prokaryotic over eukaryotic cells, a selectivity index was calculated by dividing the hemolytic concentration by the bactericidal concentration (LC99.9). A higher selectivity index indicates greater peptide selectivity toward bacterial cells over host cells.

### 5.6 Intracellular bacterial killing

Human monocytes (THP-1; TIB-202; ATCC) were cultured in RPMI 1,640 medium (Gibco) supplemented with 10% FBS. For differentiation into macrophage-like cells (dTHP-1), THP-1 monocytes were seeded in 96-well plates at 3 × 10^4^ cells/well (100 µL) in RPMI supplemented with 50 ng/mL phorbol 12-myristate 13-acetate (PMA; Sigma-Aldrich), and incubated for 48 h. The protocol for intracellular killing was adapted from Tang et al. (Tang et al., 2015) with modifications. After differentiation, dTHP-1 cells were pre-treated with one or 2 µM peptide solutions in fresh RPMI for 24 h. Subsequently peptides were removed by washing three times with PBS. An *mCherry*-expressing *S. aureus* strain, prepared as described in Section 5.2, was used. This strain is efficiently internalized by macrophages (Cuypers et al., 2024). Bacteria were added to dTHP-1 cells at a multiplicity of infection (MOI) of 2:1 and incubated for 45 min at 37 °C with 5% CO_2_ for phagocytosis. Extracellular bacteria were then removed by washing the wells three times with 60 µL PBS, followed by a final wash with 200 µL PBS.dTHP-1 cells were either lysed immediately to quantify the number of intracellular bacteria, after phagocytosis (T = 0 h) or were incubated for 24 h (T = 24 h) in RPMI supplemented with 10 μg/mL gentamicin (Sigma-Aldrich) to inhibit extracellular bacterial growth (in both peptide-treated and control dTHP-1 cells). Internalized bacteria were quantified after lysing the cells with 0.025% Triton X-100 in PBS for 15 min. Lysates were serially diluted (both at 0 h and 24 h) and plated onto BA for CFU enumeration to assess intracellular bacterial survival.

### 5.7 Bacterial endotoxin neutralization

The endotoxin-neutralizing capacity of the peptides was assessed by measuring tumor necrosis factor-alpha (TNF-α) production by dTHP-1 macrophages. dTHP-1 macrophages were stimulated with either lipopolysaccharide (LPS; *E. coli* O111:B4; 5 ng/mL, Sigma-Aldrich) or UV-inactivated *S. aureus* JAR060131 (MOI 2; as described in Section 5.2), in the presence or absence of one or 2 µM peptides. Cells were cultured in serum-free RPMI and incubated for 4 h at 37 °C with 5% CO_2_. Positive controls consisted of cells stimulated with LPS or UV-*S. aureus* without peptide treatment (no treatment, NT), and unstimulated cells served as negative controls. After stimulation, cell supernatants were collected, and cytokine levels of TNF-α were quantified using human ELISA kits (R&D Systems, USA) according to the manufacturer’s instructions. Each condition was tested in triplicate.

### 5.8 *In vitro* scratch assay: cell migration post-wounding

Cell migration following injury was assessed using an *in vitro* scratch assay with the IncuCyte wound maker tool (Sartorius). HaCaT keratinocytes were seeded in 96-well ImageLock plates (Sartorius) at 3 × 10^5^ cells/well (100 µL) in DMEM supplemented with 10% FBS and incubated at 37 °C, 5% CO_2_ for 48 h, with medium change at 24 h, until confluence. Uniform scratches were introduced using the wound maker tool according to the manufacturer’s instructions. After wounding, wells were washed twice with 100 µL PBS to remove cell debris. Cells were then treated with 1 µM peptides in serum-free DMEM, to focus specifically on cell migration from the wound edges. The absence of serum minimizes interference with peptide activity. Transforming growth factor-beta (TGF-β; human; 20 ng/mL; Sigma-Aldrich) and 5% dimethyl sulfoxide (DMSO; Sigma-Aldrich) were used as positive and negative controls, respectively. Additional controls included serum-free DMEM (no-treatment control) and 50% (*v/v*) acetonitrile in Milli-Q water as solvent control. Wound closure was monitored using the IncuCyte S3 live-cell imaging system (Sartorius) with bright-field microscopy. Images were captured every 6 h for up to 60 h. Image analysis was performed using ImageJ software (version 1.53t, NIH, USA) with the “Wound Healing” plugin, as described by Suarez Arnedo et al. (Suarez-Arnedo et al., 2020). Quantitative parameters included wound width (µm), percent wound closure, and migration rate (µm/hour).

### 5.9 *In vitro* angiogenesis assay: tube formation

Primary human venous endothelial cells (HUVECs; CSC 2V0) were used to evaluate the *in vitro* angiogenic potential of peptides using a tube formation assay, adapted from Arnaoutova and Kleinman (Arnaoutova and Kleinman, 2010). Cells were cultured in low-serum endothelial cell growth medium 2 (2% *v/v*; Promo Cell). Briefly, 96-well plates were coated with 50 µL/well of phenol red-free, growth factor-reduced basement membrane matrix (Matrigel; Corning), and incubated at 37 °C for 1 h to allow gelation. HUVECs were harvested and resuspended at a final cell density of 1.5 × 10^5^ cells/mL in serum-free endothelial cell growth medium containing a final concentration of 1 µM of peptide. Then, 100 µL of cell suspension (1.5 × 10^4^ cells/well) was added to the Matrigel-coated wells in triplicate and incubated at 37 °C, 5% CO_2_ for 8 h. Tube formation was monitored using the IncuCyte S3 live-cell imaging system (Sartorius) with bright-field microscopy. Images were captured hourly, and the 4-h time point (corresponding to optimal tube formation) was used for analysis. The number of capillary-like structures was quantified using the “Angiogenesis Analyzer” plugin in ImageJ (Carpentier et al., 2020). Each peptide condition was tested in triplicate and repeated three times using different Matrigel batches to account for batch-to-batch variability.

### 5.10 Excisional wound infection model using *ex vivo* human skin

Human skin from 3 donors was obtained via the Residual Tissue Biobank of the Red Cross Hospital (Beverwijk, Netherlands). The use of these coded, post-operative residual tissue samples was approved by patients through the informed opt-out-plus protocol of the Red Cross Hospital Skin (Code-of-Conduct-for-Health-Research, 2022) and processed for an *ex vivo* wound infection model as described (Dijksteel et al., 2020). The Wounded skin grafts were sectioned into approximately 1 cm^2^ samples using a sterile scalpel and were decontaminated by immersion in 70% ethanol for 30 min, followed by three PBS washes. Samples were placed dermis-down and wound-side-up on stainless steel grids with central indentations, positioned in 12-well plates, and cultured at the air–liquid interface in DMEM at 37 °C and 5% CO_2_. To confine reagents to the wound area, a resin barrier (OpalDam; Ultradent) was applied to the wounded area and polymerized using a hand-held UV light (Valo X) for 10 s. The wounds were then topically infected with 10 µL of *S. aureus* LUH14616 suspension of 1 × 10^7^ CFU/mL (final dose: 1 × 10^5^ CFU/graft) in PBS for 1 h, followed by topical application of 10 µL of peptide solution for 4 h. Chlorhexidine (0.5% (*v/v*) in 70% ethanol; Entick B.V.) and PBS were used as positive and negative controls, respectively. After treatment, tissue samples were transferred to polypropylene vials containing 1 mL PBS supplemented with 0.025% (*v/v*) SPS and a 7 mm stainless steel bead. Homogenization was performed using a MagnaLyser System (Roche) with three 30-s cycles at 7,000 rpm, with 30 s of cooling on ice between cycles. Homogenates were 10-fold serially diluted in PBS, and 100 µL aliquots were plated on BA plates. CFUs were enumerated after overnight incubation at 37 °C.

### 5.11 Statistical testing

All data was analyzed using GraphPad prism (version 10.2.0) and are presented as mean ± standard deviation (SD). A *p*-value of <0.05 was considered statistically significant. Statistical significance in figures is indicated as follows: *p* < 0.05 (*), *p* < 0.01 (**), and *p* < 0.001 (***), and ns denotes non-significant differences (*p* > 0.05).

## Data Availability

The original contributions presented in the study are included in the article/Supplementary Material, further inquiries can be directed to the corresponding authors.

## Abbreviations

Ahx, Aminohexanoic acid; AMP(s), Antimicrobial peptide(s); AMR, Antimicrobial resistance; CFU, Colony forming unit; DAP, 2,3-diaminopropionic acid; DAPOA, (2-(1,3-bisamino)propan-2-yl)oxy)acetic acid; DK, D-lysine; DMSO, dimethyl sulfoxide; ESKAPE(E), *Enterococcus faecium*, *Staphylococcus aureus*, *Klebsiella pneumoniae*, *Acinetobacter baumannii*, *Pseudomonas aeruginosa*, *Enterobacter* spp. (*E. coli*); Fmoc, 9H-fluorenylmethyloxycarbonyl; K, Lysine; LC99.9, Lethal concentration killing 99.9% of bacteria; LDH, Lactate dehydrogenase; MDR, Multidrug resistant; PBS, Phosphate-buffered saline; RPMI, Roswell Park Memorial Institute 1640 medium; SAAP-148, Synthetic antimicrobial and antibiofilm peptide 148; SPS, Sodium polyanethol sulfonate; TNF-α, Tumor necrosis factor-alpha; TSB, Tryptic soy broth; WST-1, Water-soluble tetrazolium salt.
