# Supplementary material for: Branched tetrameric lactoferricin peptides modified with diaminopropionic acid exhibit potent antimicrobial and wound-healing activities
Source: Front Pharmacol. 2025 Dec 2;16:1719557. doi: 10.3389/fphar.2025.1719557 (PMC12705620; doi:10.3389/fphar.2025.1719557)
Supplement: Supplementary file 1 [file Supplementaryfile1.docx]

Supplementary Material

# Supplementary Data

**Figure S1.**


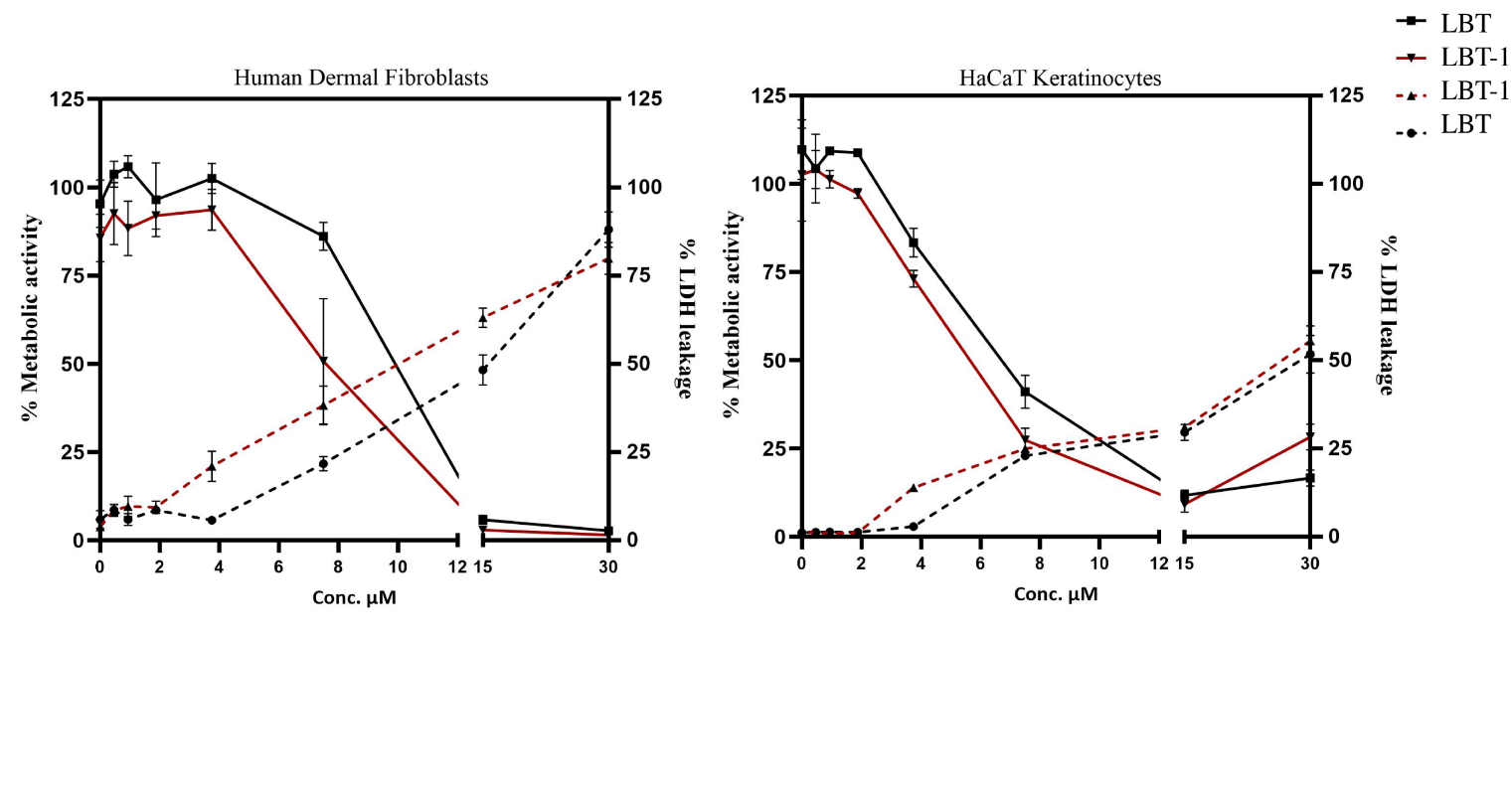


**Figure S1.** Dose-dependent cytotoxic effects of LBT and LBT-1 peptides. HaCaT keratinocytes (left panel) and human dermal fibroblasts (right panel). Metabolic activity was assessed using the WST-1 (left Y-axis; solid line), and membrane integrity was evaluated via LDH leakage (right Y-axis; dashed line). Results are presented as mean ± SD from triplicate experiments.

**Figure S2**


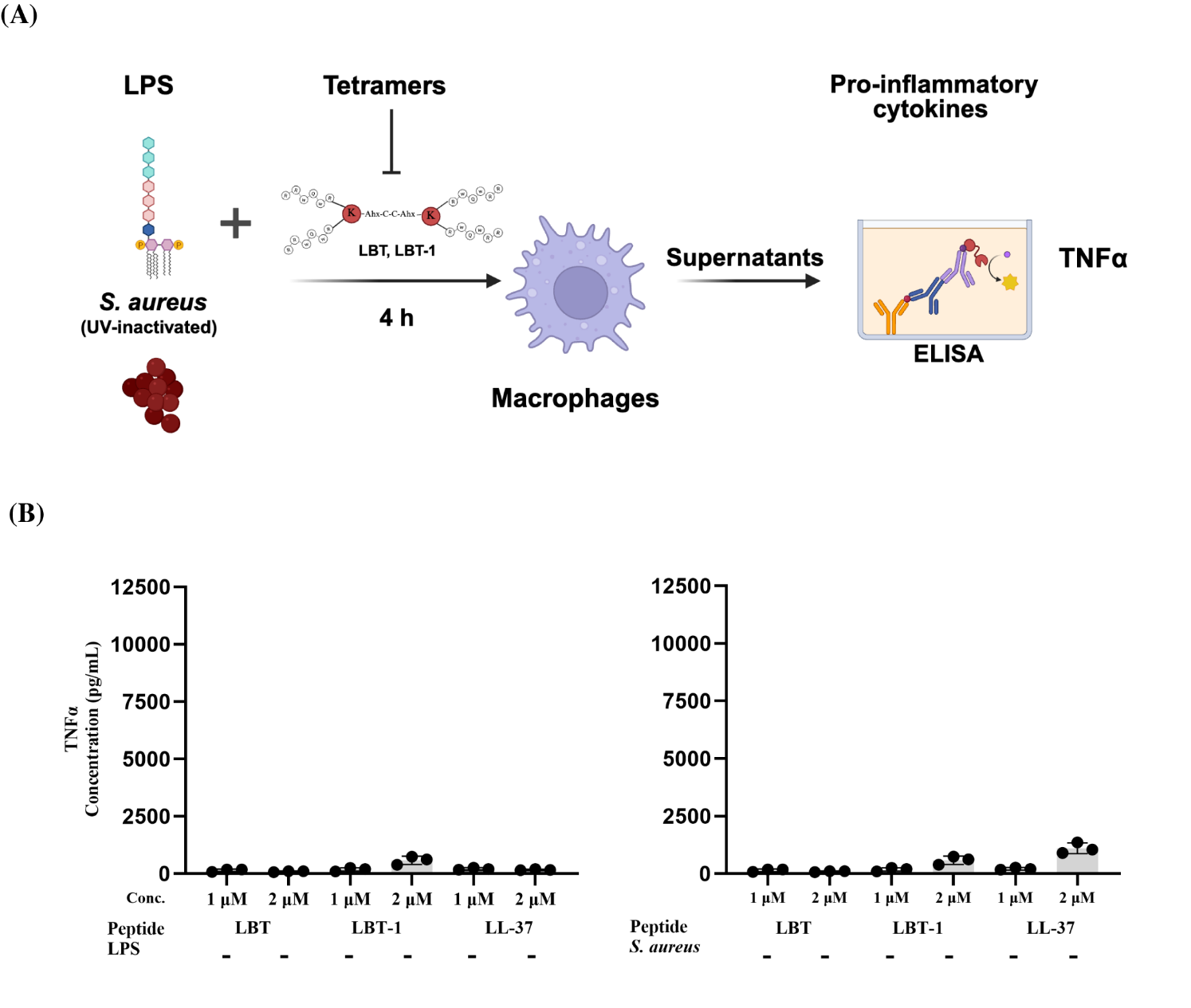


**Figure S2**(**A**). Schematic workflow of THP-1 macrophages stimulation with either LPS (*E.coli*) or UV-inactivated *S. aureus* in the presence or absence of LBT peptides is shown. Following 4 h incubation, the levels of LPS or *S. aureus* induced TNFα in the supernatants was determined using ELISA and LL-37 served as positive control. Values ± SD from triplicates. (**B**) Macrophages treated with peptides alone induced minimal or no induction of TNFɑ.
